# The Interventions and Challenges of Antimicrobial Stewardship in the Emergency Department

**DOI:** 10.3390/antibiotics12101522

**Published:** 2023-10-09

**Authors:** Jesus Ruiz-Ramos, Laura Escolà-Vergé, Álvaro Eloy Monje-López, Sergio Herrera-Mateo, Alba Rivera

**Affiliations:** 1 Pharmacy Department, Hospital Santa Creu i Sant Pau, 08025 Barcelona, Spain; amonje@santpau.cat; 2 Sant Pau Institute of Biomedical Research (IIb Sant Pau), 08025 Barcelona, Spainmrivera@santpau.cat (A.R.); 3 Infectious Diseases Department, Hospital Santa Creu i Sant Pau, 08025 Barcelona, Spain; lescola@santpau.cat; 4 CIBERINFEC, ISCIII—CIBER, Instituto de Salud Carlos III, 28029 Madrid, Spain; 5 Emergency Department, Hospital Santa Creu i Sant Pau, 08025 Barcelona, Spain; 6 Microbiology Department, Hospital Santa Creu i Sant Pau, 08025 Barcelona, Spain; 7 Genetics and Microbiology Department, Universitat Autònoma de Barcelona, 08025 Barcelona, Spain

**Keywords:** antimicrobial stewardship, drug resistance, emergency care

## Abstract

Over the last decades, we have witnessed a constant increase in infections caused by multi-drug-resistant strains in emergency departments. Despite the demonstrated effectiveness of antimicrobial stewardship programs in antibiotic consumption and minimizing multi-drug-resistant bacterium development, the characteristics of emergency departments pose a challenge to their implementation. The inclusion of rapid diagnostic tests, tracking microbiological results upon discharge, conducting audits with feedback, and implementing multimodal educational interventions have proven to be effective tools for optimizing antibiotic use in these units. Nevertheless, future multicenter studies are essential to determine the best way to proceed and measure outcomes in this scenario.

## 1. Introduction

The progressive increase in antibiotic resistance over the past decades has had a significant impact on healthcare systems worldwide [1,2,3]. It is known that excessive and inappropriate uses of antimicrobials have contributed to the generation, acceleration, and perpetuation of these multi-drug-resistant strains [4]. To minimize the development of antimicrobial resistance, successful antimicrobial stewardship programs (ASPs) have been developed in recent decades, demonstrating optimization and reduction in antimicrobial use while minimizing the generation and spread of multi-drug-resistant infections [5,6]. Consequently, the implementation of these programs has been recognized as a priority by healthcare authorities and scientific societies [7,8].

Most of the described experiences of such programs to date have focused on hospitalized patients, particularly critical patients, as well as, more recently, in the outpatient setting [9,10]. Hospital emergency departments (EDs) are particularly relevant for the implementation of ASPs. These units are where the first doses of antibiotics are prescribed in the hospital, both for incoming patients and those returning to primary care, as well as for a significant number of patients discharged directly to their homes or other healthcare facilities. Moreover, several studies have highlighted a significant increase in the number of infections caused by multi-drug-resistant bacteria in these units [11,12]. Despite guidelines recognizing EDs as key sites for ASP implementation, multidisciplinary team participation in such units remains limited [13]. Additionally, there is a lack of uniformity in ASP activities carried out in EDs, as well as in the way clinical and antimicrobial use outcomes are monitored [14,15]. In this review, we highlight key elements to consider when implementing ASP activity in EDs.

## 2. Multi-Drug-Resistant Bacteria in Emergency Departments

The widespread increase in antimicrobial resistance complicates the choice of appropriate empirical treatment, with a direct impact on the morbidity and mortality of patients, especially in cases of sepsis [16]. EDs present specific challenges for monitoring bacterial resistance and adopting measures to prevent its spread due to its inherent characteristics: the need for rapid care, short stays, and, in most cases, discharge of patients to their homes with empirical treatment for unidentified pathogens [17]. Moreover, up to 80% of hospitalized patients enter through EDs, underscoring the crucial role of this service in introducing multi-drug-resistant microorganisms to the hospital. On the other hand, empirical treatment initiated in these units often continues in hospital wards, significantly impacting antimicrobial consumption [17].

Several epidemiologic surveillance studies have shown an increase in the prevalence of infections caused by multi-drug-resistant strains. A longitudinal surveillance study conducted in several US hospitals from 2012 to 2017 showed a decrease in the incidence of infections caused by methicillin-resistant *Staphylococcus aureus* (MRSA), vancomycin-resistant *Enterococcus* spp., carbapenem-resistant *Acinetobacter* spp., and multi-drug-resistant *Pseudomonas aeruginosa*. The incidence of infections caused by carbapenem-resistant Enterobacterales did not change, but the incidence of infections caused by extended-spectrum β-lactamase (ESBL)-producing strains increased by 53.3%, especially due to a rise in community-acquired infections [18]. In the European Centre for Disease Prevention and Control (ECDC) surveillance 2021 report, an increase was observed in the percentage of carbapenem-resistant *E. coli* and *K. pneumoniae* strains, vancomycin-resistant *Enterococcus faecium*, and carbapenem-resistant *Acinetobacter* spp., as well as an increase in strains of *Streptococcus pneumoniae* with reduced susceptibility to penicillin (14% in 2017 to 16% in 2021) [19]. Furthermore, there has been a dramatic increase in the prevalence of Gram-negative bacteria producing narrow spectrum β-lactamase CTX-M in recent decades, replacing TEM and SHV variants as the most frequent type of ESBL. The blaCTX-M genes have migrated to highly transmissible plasmids associated with the community circulation of ESBLs [20].

In healthcare units (HUHs), despite having a limited number of records, a significant increase in infections caused by multidrug-resistant strains has been observed. A multicenter study conducted in HUHs in the United States between 2018 and 2019 found a prevalence of ESBL-producing strains in urinary tract infection (UTI) patient samples of 17%, ranging from 5% to 45%, depending on the region studied. Resistance rates to other antibiotics were 32.3% for fluoroquinolones, 13.7% for gentamicin, 1.3% for amikacin, and 0.3% for meropenem [21].

Another retrospective study of all patients presenting with febrile UTIs at HUHs in 21 different centers in the US between 2017 and 2019 found that of the 4107 included patients, 530 (12.9%) had infections caused by *Escherichia coli*, *Klebsiella pneumoniae*, or *Proteus mirabilis* that were resistant to third-generation cephalosporins. In this patient group, empirical antibiotic treatment was discordant in 63% of cases, compared to 7% of controls without resistance (OR 21.0; 95% CI 16.9 to 26.0). They also had a longer hospital stay (adjusted mean difference of 29.7 h; 95% CI 19.0 to 40.4) and higher 90-day mortality (12% in patients with resistance versus 8% in controls, adjusted OR 1.56; 95% CI 1.07 to 2.28) [22].

Regarding ED, there are limited published studies regarding variations in the resistance profile, specifically in these units, which will be a key element for improvement in the coming years. However, some studies have shown a progressive increase in infections by ESBL-producing Enterobacteriaceae in the case of urinary infections and bacteremia [23,24], which will be the main challenge for ASP in the coming years.

A multinational survey of non-hospitalized patients with infections caused by ESBL-producing Enterobacterales identified risk factors of recent antibiotic use; these included residing in a long-term care facility, recent hospitalization, age of 65 years or older, and male gender [25]. Another study identified hospitalization, long-term care, antibiotic exposure in the previous 90 days, and isolation of a fluoroquinolone- or ceftriaxone-resistant strain in the previous year as risk factors [26].

Although several studies have attempted to develop clinical tools to predict the risk of infection by multidrug-resistant Enterobacterales [25,26,27], the results obtained lack specificity, as some community-acquired UTI patients caused by ESBL-producing Enterobacterales do not exhibit risk factors that imply the selection of appropriate empirical treatment [22]. In fact, in Ben-Ami et al.’s multinational study, 34% of ESBL-producing strain isolates (115 out of 336 strains) had no identified contact with the healthcare system [20].

Given all of the above, the appropriateness of empirical antibiotic treatment in EDs is becoming increasingly complex in an environment with constantly rising resistance rates. Physicians working in these units are aware to varying degrees of this issue, and they complain of a lack of feedback on antibiotic prescriptions and knowledge about local resistance rates. In this sense, artificial intelligence models should play a key role in EDs in the coming years, helping to predict the risk of multidrug resistance in patients treated in EDs [28].

## 3. Antimicrobial Prescription in Emergency Departments

The EDs are an important setting for evaluating inappropriate antibiotic prescribing practices, given that they exist at the intersection of the community and the hospital. The EDs also present unique challenges for appropriate antibiotic prescribing, as clinicians often encounter diagnostic uncertainty and time constraints. Various studies have assessed the percentage of inappropriate antibiotic prescriptions in the ED, yielding divergent results ranging from 25% to 50% [29,30,31]. The variability in the definitions used to delineate this wide range can account for much of this inconsistency. Nevertheless, all studies indicate that there is substantial room for improvement in antimicrobial prescription practices in the ED, although their impact on the development of resistance and clinical outcomes remains uncertain.

## 4. Antimicrobial Stewardship Indicators in Emergency Departments

Despite various scientific societies positioning EDs as preferred points for the implementation of ASPs [32,33], there still exists a lack of uniformity in the types of indicators used to monitor ASP activities in these units. Differences in ED operations, the diversity of treated pathologies and patient profiles, limitations of information systems, and loss of patient follow-up after discharge hinder their implementation.

Over the past few years, studies describing ASP experiences in EDs have utilized diverse indicators. A recent systematic review of 26 studies categorized the ASP indicators used in EDs into four categories: antimicrobial consumption indicators, microbiological indicators, process indicators, and outcome indicators. It concluded that the high heterogeneity of indicators used in EDs makes monitoring these programs challenging, emphasizing the need to standardize indicators for optimizing antimicrobial use in these units [34]. Recently, Ruiz et al. [35], using a modified Delphi methodology, attempted to standardize the prioritized indicators for ASP implementation in these units, establishing different levels of priority for their adoption.

Based on these reviews, ASP activities in EDs should primarily focus on reducing antimicrobial consumption and improving health outcomes, similar to hospital-based ASPs. However, the impact of ASP activities in EDs on microbiological indicators and resistance reduction remains uncertain.

Difficulties exist that hinder indicator standardization. Firstly, not all hospitals have the necessary informatics tools to measure antimicrobial consumption in these units, making it challenging to assess its magnitude in the ED and, more importantly, to determine if a direct reduction is achieved. Another question is what is the most suitable unit of measurement for antimicrobial consumption in EDs. Since these services have high patient turnover and do not generate hospital stays, the percentage of patients receiving antibiotics in the ED, or the defined daily dose (DDD) per 1000 patients, has been the most commonly employed indicator [36]. Another unit of measurement that could be considered is the number of prescriptions per 1000 inhabitants for those antibiotics and infections that do not require admission and are managed on an outpatient basis.

Another aspect to consider is which group of antibiotics would be a priority for monitoring their consumption. Due to their impact on resistance generation, relevant indicators would focus on carbapenems and fluoroquinolones due to their high impact on cross-resistance to other antibiotics; aminoglycosides due to their high nephrotoxicity and ototoxicity; and finally, coverage of anti-MRSA agents. It is also advisable to monitor antimicrobial consumption from the perspective of the most prevalent infectious syndromes. Some relevant indicators could include the percentage of patients diagnosed with pneumonia treated with quinolones, the percentage of patients treated with antibiotics for exacerbation of chronic obstructive pulmonary disease (COPD), the percentage of multi-resistant-organism-caused UTIs, or the percentage of patients with skin and soft tissue infections receiving anti-MRSA coverage [34].

The World Health Organization (WHO) created a new classification in 2017 in which antibacterial medicines were stratified into three groups, Access, Watch, and Reserve (AWaRe), based on their spectrum, anticipated risk of resistance development, risk of toxicity, and clinical utility; this was reviewed in 2021 [37]. The monitoring of antibiotics in the emergency department should consider the risk of antibiotic resistance described in this document. A recent systematic review found that more restricted utilization of “Watch” and “Reserve” agents such as carbapenems, third-generation cephalosporins, or quinolones might be greatly beneficial [38]. Most common ED infections can be either treated with no or Access antibiotics. It should be noted that all antibiotics, irrespective of AWaRe category, could be associated with antibiotic-resistant strain selection, indicating the need to enhance focus on symptomatic care for minor infections with no routine antibiotic treatment.

On the other hand, to achieve better health outcomes, the most relevant interventions would be those that demonstrate a decrease in the number of return visits to the ED or infection-attributed mortality. An interesting measure is the review of cultures from discharged patients to verify if the microorganism is susceptible to the prescribed antibiotic.

## 5. Antimicrobial Stewardship Interventions in the Emergency Department

**Figure 1:**
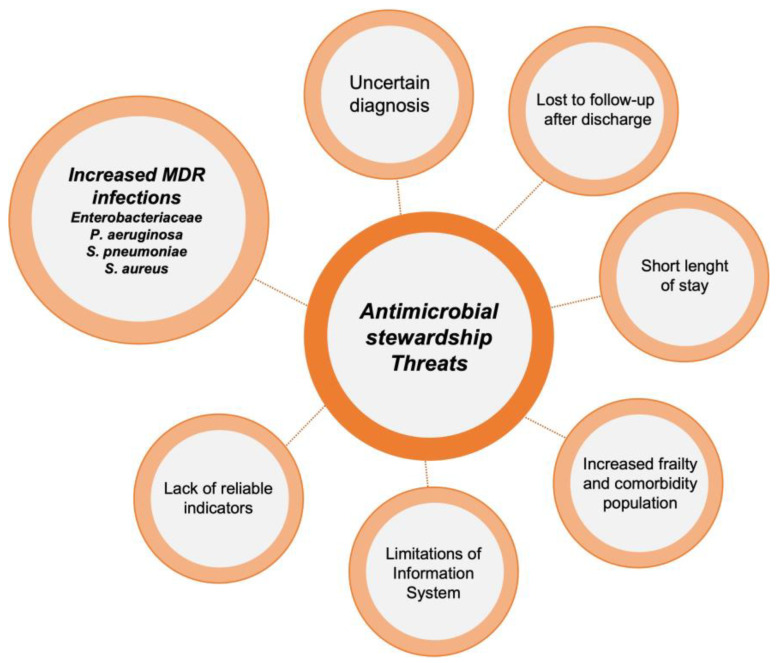
Threats to the implementation of antimicrobial stewardship programs in emergency departments.

**Figure 2:**
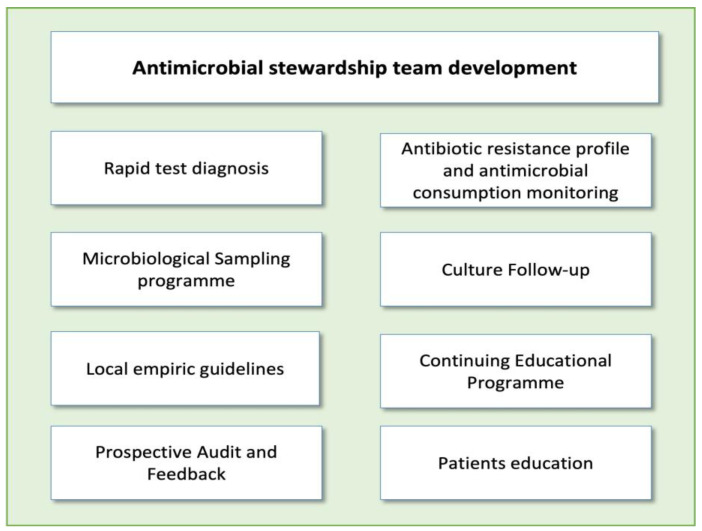
Main antimicrobial stewardship interventions in emergency departments.

Implementing ASP activities in EDs poses a significant challenge. The unique characteristics of this unit, including high patient and staff turnover, make it difficult to carry out many of the ASP activities typically applied in hospitalized patients, such as de-escalation of antibiotic treatment or optimization of duration based on patient evolution. It should be noted that each intervention requires a personalized approach, evaluating potential barriers and facilitators before execution. However, there are several activities that have proven highly effective in optimizing antimicrobial use in these units [14]. The main threats for ASP implementations in EDs and the most popular interventions described in these units are reflected in Figure 1 and Figure 2.

### 5.1. Formation of an Antimicrobial Stewardship Team in EDs

Establishing a multidisciplinary team composed of emergency physicians, infectious disease specialists, pharmacists, microbiologists, and nurses is a crucial first step in successfully implementing an ASP in the ED. ASP efforts must be multidisciplinary, collaborative, patient-centered, and have full support from hospital administrators. The role of a leader in the ED is a key figure, serving as a link between ASP team leadership and frontline clinicians to facilitate intervention implementation and provide bidirectional feedback.

ED pharmacists must play an essential role in ASP implementation in these units. Pharmacists play an essential role in the establishment and implementation of ASPs across inpatient and outpatient settings. Through regular review of antibiotic regimens as part of prospective audits with intervention and feedback, they have the opportunity to optimize antimicrobial selection, dose, and duration. Participation in empiric ED local guidelines considering antimicrobials included in hospital formulary is also an important role of clinical pharmacists. Furthermore, they have the necessary skills to track and report ASP metrics. Several studies have found the ASP incorporating clinical pharmacist in the ED optimize antimicrobial therapy and improves patients’ outcomes [39,40].

ED ASP teams are greatly enhanced by the support of other key groups in hospitals, including clinicians and department heads (as they play an essential role in ensuring clinicians are fully engaged in and supportive of efforts to improve antibiotic use). Infection preventionists and hospital epidemiologists (monitoring and prevention of healthcare-associated infections), Drug and Therapeutics Committees (providing and approving local guidelines to ensure rational antibiotic use, ensuring adequate availability and restrictions on the use of antimicrobials in the hospital pharmacotherapy guide), quality improvement staff (as optimizing antibiotic use is a medical quality and patient safety issue), information technology staff (integrating stewardship protocols into existing workflow, creating prompts for action to review antibiotics, and facilitating the collection of antibiotic use data), and nurses (who can ensure an appropriate culture obtention antibiotic as part of administration) [41].

### 5.2. Rapid Diagnostic Tests

Conventional microbiological methods based on culture and antimicrobial susceptibility testing might require 24–72 h or even longer to yield results; thus, additional approaches are imperative to expedite the diagnostic process. This need is especially relevant within EDs, where a combination of diagnostic uncertainty and time constraints, coupled with the rise in antimicrobial resistance, often lead to inadequate empirical treatment [42].

Rapid diagnostic tests (RDTs) for infectious diseases enable the acceleration of pathogen identification and the determination of antimicrobial resistance patterns, which is of vital importance for structuring appropriate targeted antimicrobial therapy [33,34]. Likewise, the rapid detection of multi-resistant or highly transmissible microorganisms enables the implementation of control measures to restrict their transmission, a fact that has become especially clear with the emergence of SARS-CoV-2. The utility of RDTs in reducing turnaround time has been highlighted in several studies, but their implementation without the support of an ASP has yielded modest advantages in terms of optimizing antimicrobial therapy and impacting patient outcomes [43,44,45,46,47,48,49]. The use of RDTs requires the multidisciplinary involvement of microbiologists, physicians, and pharmacists to ensure that the indication is appropriate and that the result information is properly interpreted by the responsible clinician to guarantee its usefulness in optimal patient management [43,44].

Although a specific definition of RDTs has not been established, they are generally understood to provide results to the clinician within 4–6 h [50]. Several technologies have been employed for RDTs for infectious diseases in the ED, including immunoassay-based tests, mass spectrometry, single and multiplex nucleic acid amplification, microarrays, and fluorescent in situ hybridization (FISH) [51,52]. These methods focus on detecting either single or multiple pathogens most commonly associated with infectious syndromes like bloodstream infections, meningitis/encephalitis, respiratory tract infections, sexually transmitted infections, or gastrointestinal infections. Some commercially available rapid diagnostic platforms also enable the detection of antimicrobial resistance genes in the same assay.

In the case of bloodstream infections, it should be noted that most of the currently available approaches to speed up the diagnosis for pathogen identification and antimicrobial susceptibility testing are performed after blood culture positivity [53]. The benefits of RDTs in decreasing the time to optimal antimicrobial therapy have been demonstrated in several studies, but their impact on patient outcomes such as mortality or hospital length-of-stay has shown variable results [54]. This variability has been associated with several factors, such as the existence of an ASP, the study design, or the population selected. A meta-analysis demonstrated decreased mortality risk from bloodstream infections with RDTs (only for pathogen identification) together with ASPs compared with conventional microbiological methods (OR, 0.64; 95% CI, 0.51–0.79) [46]. Other studies comparing rapid identification and antimicrobial susceptibility testing versus conventional methods failed to demonstrate differences in patient outcomes [55,56]. In addition, a Cochrane review revealed no association between RDTs and antibiotic susceptibility testing to reduce mortality or shorten the length of stay [57].

It is worth mentioning that most of the existing literature about the clinical impact of RDT on bloodstream infections has not focused specifically on ED patients. Nonetheless, the ideal scenario would be to perform direct testing from whole blood [58]. Currently, there is only one Food and Drug Administration-cleared assay that can directly use whole blood, the T2 Bacteria, T2 Candida, and T2 resistance Panels (T2 Biosystems, Lexington, MA, USA), which is a magnetic-resonance-based molecular assay with a turnaround time of 3–5 h. Emerging technologies based on microfluidics, nanotechnology, next-generation sequencing, and CRISPR-based diagnostics seem to provide promising results in the rapid identification of pathogens and antimicrobial resistance genes from whole blood samples [52,58]. More data are required to determine the clinical value and cost-effectiveness of these approaches.

Urinary tract infections (UTIs) are a common indication for antibiotic prescriptions in ED. Traditional microbiological diagnosis relies on microscopic sediment examination, culture, and antimicrobial susceptibility testing [59]. Emerging rapid diagnostic methodologies, including biosensor-based platforms and microfluidics, are being developed to address the diagnostic delays caused by the standard 48 h turnaround time for urine culture results. It is important to mention that a key component of ASPs for UTIs in ED is reducing the number of unnecessary urine cultures ordered [60]. In the ED, urine cultures are often ordered for a variety of indications, and a positive urine culture for patients with nonspecific UTI symptoms can be a misleading finding, which can lead to unnecessary antimicrobial treatment [61].

For respiratory tract infections (RTIs), RDTs could play a crucial role in distinguishing between bacterial and viral infections and consequently aid in the prevention of antibiotic use for viral infections. Rapid immunoassays are widely used in the ED for virus (FLU A and B virus, RSV, and SARS-CoV-2) and bacterial pathogens (*Streptococcus pneumoniae* and *Legionella pneumophila* serogroup 1) detection. Several molecular tests are also commercially available, encompassing pathogen-specific PCR assays and multiplex assays that simultaneously test viral and bacterial respiratory pathogens [62]. A systematic review and meta-analysis assessing the diagnostic test accuracy of RDTs in ED and other community settings found that the best performance is achieved with molecular tests, unlike rapid antigen-based detection tests, which exhibit high false-negative rates [63]. The authors also demonstrated the poor diagnostic accuracy of clinical signs and symptoms and biomarkers as stand-alone indicators, while lung ultrasound shows high sensitivity and specificity for the diagnosis of bacterial pneumonia compared with chest X-ray. The impact of RDTs on management, reducing unnecessary treatment, and outcomes in patients with clinical signs and symptoms of respiratory tract infection during their ED visits have been evaluated in several studies. In a single-hospital-based study, the use of a rapid syndromic PCR panel in patients presenting to the ED with suspected community-acquired pneumonia was associated with a significant reduction in time to results and increased detection of relevant pathogens compared to standard diagnostic methods [64]. A randomized controlled trial of pediatric and adult patients attended to in the ED with symptoms of acute RTI found that a rapid multiplex PCR was associated with a trend towards decreased antibiotic use, but there was no difference in outcomes, antiviral use, or LOS [64]. A recent meta-analysis conducted in ED and inpatients found that rapid multiplex PCR testing for respiratory viruses was associated with reduced time to results and LOS, improved antiviral use, and infection control in influenza patients [65]. RDTs for acute pharyngitis, one of the most prevalent infections, should also be considered as one of the most efficient strategies to minimize the use of antimicrobials in respiratory infections treated in ED. It is well known that only a reduced part of these infections has a bacterial etiology, with most cases caused by group A beta-hemolytic streptococci (GABHS). RDTs for GABHS detections have become a popular alternative to throat swab cultures. The issue of poor RDT still hampers screening and treatment of GAS pharyngitis in the ED. Despite poor RDT sensitivity, evidence for the utility combined with clinical scores to reduce antibiotic use for GAS pharyngitis in pediatric EDs is growing [66].

For other infections presenting at the ED, such as central nervous system infections and sexually transmitted infections, the analytical performance and clinical impact of RDTs are also well documented [67,68]. A multiplex PCR for meningitis/encephalitis has demonstrated improved diagnostic yield and turnaround time in several studies of suspected community-onset CNS infection with a reduction in length of stay and improved antimicrobial therapy [69,70]. For sexually transmitted infections, the use of rapid molecular tests for the detection of *Neisseria gonorrhoeae* and *Chlamydia trachomatis* in ED has been associated with a significant increase in antimicrobial treatment appropriateness, faster turnaround results, and lower costs [71,72].

RDTs have several drawbacks that must be taken into mind, such as cost, the inability to distinguish between viable and non-viable microorganisms, and the detection only of the microorganisms and resistance genes specifically targeted by the assay. In addition, there is also the possibility of detecting resistance genes that do not translate into phenotypic resistance due to differences in expression [73]. Despite the usefulness of RDTs, for now, they cannot displace traditional gold standard culture-based techniques, which remain necessary for determining antimicrobial susceptibility.

### 5.3. Culture Follow-Up Programs

Most patients treated in EDs are discharged with empirical antibiotic therapy for infection management, often without a definitive microbiological result. Therefore, one of the fundamental ASP functions in EDs is to have specific follow-up programs for patients after their discharge from the unit. Follow-up culture programs upon ED discharge with treatment adjustment afterward are among the most effective measures to implement in EDs. Experiences with these programs have demonstrated the ability to optimize treatment for conditions like urinary tract infections and bacteremia, resulting in reduced return visits to the ED and cost savings [74,75,76].

### 5.4. Availability of Resistance Profiles and Antimicrobial Consumption Data

The availability of local resistance profiles specific to the ED is a key element within ASPs in these units, as it allows for the assessment of resistance and modification of empirical therapy guidelines. Antibiograms in these units face certain challenges for extraction depending on available information systems, including their separation from the rest of the hospital data, their association with specific diagnostic processes, and the elimination of duplicate data. These resistance profiles should be used to guide unit-specific recommendations for the empirical treatment of all common bacterial infections. On the other hand, trends in antimicrobial consumption data in the unit help identify patterns of inappropriate use that can be corrected through educational interventions.

### 5.5. Education for Patients and Professionals

Although training healthcare professionals in the appropriate use of antimicrobials is a cornerstone of ASPs in any setting, there is extensive discussion on how to carry out this activity. Traditional educational sessions have shown limited effectiveness in producing lasting changes in clinical practice. While favorable studies with educational interventions alone have been published, it is more common to see education included as part of a set of interventions [77,78]. In such interventions, clinical decision support systems play an essential role and have been shown to improve prescription appropriateness in EDs [79,80].

Multidimensional strategies offer a deeper solution to achieving ASP goals. These types of interventions involve various joint actions associated with educational intervention, including the implementation of local treatment guidelines or audits before and after their application, with or without feedback. Multifaceted control interventions have been shown to improve antibiotic prescription in EDs for specific conditions such as pneumonia, UTIs, or skin and soft tissue infections [81,82].

Training activities on the appropriate request for microbiological samples should be part of the ASPs activity in EDs. The utility of obtaining blood cultures in the ED setting has been questioned due to frequent false positive results from contamination, low positivity rate, and lack of impact on management in uncomplicated bacterial infections. Eliminating blood cultures in immunocompetent patients with common illnesses such as urinary tract infections, community-acquired pneumonia, and cellulitis significantly reduces the number of blood cultures, producing substantial savings. This needs prospective study and validation.

Training activities for ED physicians should also consider essential aspects of infection management, such as adequate control of the infectious focus. Source control significantly improves outcomes compared to early and effective antimicrobial therapy and should never be assumed to be adequately addressed by broad-spectrum agents. Because the effectiveness of source control depends on timing, it is essential to perform appropriate procedures as quickly as possible for septic patients. Both surgical site infection and intraabdominal infection guidelines emphasize the absolute necessity of source control to improve patients’ outcomes. Prioritizing source control also helps clinicians determine the duration of antibiotic treatment [83]. Therefore, it is a key task of the infectologists and emergency physicians of ASP teams to advise the ED physicians responsible for the patients on this point.

Educational practices towards patients include local prescriber activities and community-based efforts. Studies evaluating patient education often include passive educational leaflets and focus most often on the appropriate treatment of upper respiratory tract infections. Given the high number of patients treated in EDs, these units can play a key role in this training work, and some successful experiences in them have been described [84]. Future studies should evaluate the impact of educational interventions, including providing education to non-prescribers and disease states beyond respiratory tract infections.

### 5.6. Inclusion of Biomarkers

The use of biomarkers to reduce antimicrobial use has also been studied within ASPs in EDs, with particular relevance to the use of procalcitonin (PCT). Schuetz et al. [85], in a meta-analysis of 32 studies in primary care, ED, and ICU settings, found a reduction in total antibiotic exposure from 8.1 days to 5.7 days when comparing usual care to a PCT-based algorithm. Mathioudakis et al. [86], in another meta-analysis of patients with exacerbations of chronic obstructive pulmonary disease, observed a decrease in antibiotic prescriptions. However, several studies have yielded negative results, casting doubt on the use of this biomarker [87]. Therefore, the implementation of this strategy in the pursuit of reducing antibiotics in EDs should be accompanied by audit and feedback with the prescriber that reinforce diagnostic uncertainty, as the mere inclusion of the test within the ED is unlikely to lead to a change in antibiotic use in the unit if not coupled with ASP guidance.

### 5.7. Formulary Restrictions

The antimicrobial treatment selected by ED physicians affects subsequent therapy options for both hospitalized and ambulatory patients. Limiting the use of certain broad-spectrum antibiotics in EDs has been proposed as a strategy to avoid the indiscriminate use of certain antimicrobials to ensure the efficacy of these agents over time. Among these methods, excluding certain antibiotics from the ED’s formulary or requiring prior authorization from the infectious diseases service of the center is an alternative implemented in many centers [88]. However, this option presents several relevant limitations. Cumulative evidence to date suggests that antibiotic cycling has limited efficacy in preventing antibiotic resistance, and its clinical impact in severe patients with a high risk of MDR infection remains controversial [89].

## 6. Outcomes of ED Antimicrobial Stewardship Programs

To date, there are few high-quality studies on the effectiveness of ASPs in EDs, with most of the described experiences being single-center descriptive studies with limited analysis of clinical evolution and resistance generation [14,40].

The implementation and adaptation of clinical guidelines and protocols associated with ASPs have been mainly described for respiratory infections and UTIs, yielding mixed results regarding antibiotic reduction. Angoulvant et al. [90] demonstrated a significant change in antibiotic prescription trends in seven pediatric EDs following the implementation of national empirical treatment guidelines for respiratory infections. On the other hand, Akenroye et al. [91], comparing outcomes before and after implementing a protocol for bronchiolitis, observed improvement in diagnostic test ordering without a significant reduction in antibiotic use.

The effectiveness of interventions seems to be related to the intensity of the intervention, primarily of an educational nature. Buising et al. [92] compared three different strategies over three different time periods: a first episode of delivering treatment guidelines to prescribers, a second episode incorporating educational activities, and a third episode introducing an electronic prescription support system with hospital guidelines. The risk of receiving guideline-concordant treatment significantly increased in the second (OR: 2.79 (1.88–4.14)) and third (OR: 1.99 (1.07–3.69)) periods compared to the first period.

Marrie et al. [93], in a clinical trial comparing conventional management versus an intervention protocol for pneumonia based on clinical guideline recommendations, showed a decrease in length of stay, hospitalization rates, and duration of intravenous antibiotic therapy. Metlay et al. [94] observed a 10% reduction in antibiotic prescriptions for acute respiratory infections in the intervention group in a clinical trial across 16 hospitals, including audit and feedback interventions and educational material delivery to clinicians and patients, which included posters, educational brochures, and a waiting room video. Borde et al. [95] implemented an ASP aimed at reducing cephalosporin and fluoroquinolone prescriptions in the ED. They reviewed and updated local guidelines for the most common infections, conducted an educational activity on antimicrobial use, and promoted consultation with infectious disease units, resulting in a reduction in daily antibiotic doses, especially for cephalosporins.

In the case of ASPs focused on UTIs, the main objectives have been centered on reducing antibiotic treatment for conditions with positive urine isolation without infection, including asymptomatic bacteriuria, facilitating appropriate diagnosis and selection of appropriate antimicrobial therapy, specifically with a reduction in quinolones for uncomplicated cystitis. Studies have focused on including educational interventions for prescribing physicians, with specific recommendations for microbiological sample collection and local guidelines based on institutional antibiograms. In a study that included the development of specific UTI guidelines based on institutional resistance patterns, recommendations for sample collection, and feedback to physicians, Hecker et al. [96] observed improved adherence to UTI guidelines with a 30% decrease in fluoroquinolone use for uncomplicated cystitis. Percival et al. [97] showed a 40% increase in adherence to UTI guidelines, including a specific educational program within the institution and educational material delivered via email, along with a retrospective audit and feedback to prescribers.

## 7. Conclusions

In a context of increasing resistance, EDs are crucial settings for implementing ASPs. However, the unique characteristics of these units hinder the implementation of many typical ASP practices. Although most published studies show improvement in the appropriateness of antimicrobial prescribing following implementation, the heterogeneity of interventions performed and the outcome indicators used make it challenging to select activities for implementing ASPs in EDs. Nevertheless, improving the diagnostic process, post-discharge culture follow-up and monitoring of resistance and antibiotic consumption should be part of these interventions. Each service/department should approach the implementation of these programs according to the institution’s characteristics, volume, environment, and workflow.

## Data Availability

The data presented in this study are available within the article or upon request from the corresponding author.
